# Silver Linings Reported by Australians Experiencing Public Health Restrictions during the First Phase of the COVID-19 Pandemic: A Qualitative Report

**DOI:** 10.3390/ijerph182111406

**Published:** 2021-10-29

**Authors:** Narelle Campbell, Sandra C. Thompson, Anna Tynan, Louise Townsin, Lauren A. Booker, Geoff Argus

**Affiliations:** 1 Rural and Remote Health College of Medicine and Public Health, Flinders University, Darwin 0815, Australia; 2 Western Australian Centre for Rural Health, University of Western Australia, Geraldton 6530, Australia; sandra.thompson@uwa.edu.au; 3 Research Support Team, Darling Downs Health, Toowoomba 4350, Australia; anna.tynan@health.qld.gov.au; 4 Southern Queensland Rural Health, The University of Queensland, Toowoomba 4350, Australia; g.argus@uq.edu.au; 5 Research Office, Torrens University Australia, Adelaide 5000, Australia; louise.townsin@torrens.edu.au; 6 La Trobe Rural Health School, University Department of Rural Health, La Trobe University, Bendigo 3551, Australia; l.booker@latrobe.edu.au; 7 School of Psychology and Counselling, University of Southern Queensland, Toowoomba 4350, Australia

**Keywords:** mental health, resilience, policy, rapid evaluation, theme identification

## Abstract

This national study investigated the positives reported by residents experiencing the large-scale public health measures instituted in Australia to manage the first wave of the COVID-19 pandemic in 2020. Most Australians had not previously experienced the traditional public health measures used (social distancing, hand hygiene and restriction of movement) and which could potentially impact negatively on mental well-being. The research design included qualitative semi-structured phone interviews where participants described their early pandemic experiences. Data analysis used a rapid identification of themes technique, well-suited to large-scale qualitative research. The ninety participants (mean age 48 years; 70 women) were distributed nationally. Analysis revealed five themes linked with mental well-being and the concept of silver linings: safety and security, gratitude and appreciation, social cohesion and connections, and opportunities to reset priorities and resilience. Participants demonstrated support for the public health measures and evidence of individual and community resilience. They were cognisant of positives despite personal curtailment and negative impacts of public health directives. Stories of hope, strength, and acceptance, innovative connections with others and focusing on priorities and opportunities within the hardship were important strategies that others could use in managing adversity.

## 1. Introduction

While infectious disease epidemics have occurred throughout history, developed countries have progressively focussed on reducing the burden of chronic disease, including mental health [1,2]. This changed radically in 2020. Even with the marvels of modern science and communications, the COVID-19 pandemic progressed with a speed that has challenged all countries and their populations.

Australia’s response was largely based upon time-honoured public health approaches to controlling communicable diseases; quarantine and restriction of people’s freedom of movement [3]. Specifically, restrictions imposed early in the pandemic included the closure of international borders, state and territory border closures, limitations on the movement and numbers allowed at social gatherings, closure of businesses with only those deemed essential allowed to stay open, and the requirement for people to work from home where possible. In addition, public health messaging focused on personal physical distancing and hygiene practices [4]. Between mid-March and May 2020, workers were required to work from home where possible and many “non-essential” businesses were prohibited from trading. Many school students were required to study from home unless their parents were “essential” workers [5]. Thus, parents could be simultaneously working from home and assisting their children to study. The government introduced a range of economic measures, such as Jobkeeper and Jobseeker, for businesses and individuals impacted by the pandemic [6]. Despite these financial supports, household finances were reported to have worsened for one in three households during the first phase of the pandemic in Australia [7]. Family members were sometimes separated and confined to their homes, some experiencing stress, anxiety and mental health issues [8,9]. Some people endured severe financial stresses, with others experiencing or concerned about job losses [7]. Compared to many international efforts, Australia’s response was effective in reducing infection rates [4,10], however disruptions to daily life were considerable. Perceived vulnerability and social isolation [9] during the early stages of the pandemic response inevitably impacted on individuals, families, and the community.

In Australia, one in five people report having a mental illness [11]. Mental and substance use disorders contributed 12% of Australia’s total burden of disease in 2015, making it the fourth highest disease group contributing to total burden [12]. One common finding is that people who suffer from a chronic disease are more likely to also suffer from depression or other mental illness [13,14]. Imposed quarantine or isolation is unfamiliar and unpleasant. While all people are at risk of psychological harm when kept in isolation, the most vulnerable in these situations are children and adolescents, older adults, minority groups, those from lower socio-economic groups, females, and people with pre-existing mental health conditions [15,16,17]. Many people in vulnerable groups rely on supports and programs that may be lost or interrupted due to restrictions associated with COVID-19 [18].

Yet even in the midst of difficulties and challenges, many people show strength and resilience in overcoming hardship and the ability to see the positives and adapt to future challenges [19]. The current study highlights previously unreported positive perspectives of Australians during the early months of implementation of public health measures and restrictions to help control COVID-19 in Australia in the first year of the pandemic. Understanding the perceived “silver linings” reported by people with chronic disease during the initial lockdowns in Australia may be important for promoting health during a pandemic. Future measures that support better mental health outcomes despite the restrictions may be guided by knowledge about the adaptability and resilience of people experiencing the restrictions. Therefore, the research question forming the basis of this analysis was “what unexpected positives or silver linings were reported by Australians during the first phase of the pandemic?”

## 2. Methods

### 2.1. Study Design

This study is part of a large quantitative–qualitative exploratory study investigating the experiences of Australians during the first phase of the pandemic, and the methods have been previously reported [20]. For the qualitative component, semi-structured interviews probed a series of topics about attitudes to the public health policy measures. It also asked participants about their experience of the pandemic and its impact on their everyday life, including changes they had made, changes in their community, positives observed from the pandemic (“Do you think that positives have emerged from the COVID-19 pandemic? If so, what are they?”) and the pandemic’s apparent impact on their future. One of the themes that emerged as data collection and analysis proceeded was termed “silver linings”, which focussed on understanding how participants reflected on and made sense of the changes and unexpected positives during this challenging time.

Interviews were undertaken by a team of 20 researchers from centres which routinely collaborate through the Australian Rural Health Education Network (ARHEN). A written protocol and training ensured interviews were conducted by the interviewers using a standard approach. The protocol also contained instructions around notating and tagging the interviews at time intervals during the interview in a manner consistent with the rapid identification of themes from audio recordings (RITA) approach [21]. The tags had previously been agreed to by the researchers and resulted in a searchable database of the interviews.

### 2.2. Study Participants and Interview Data Presentation

Participants had completed a national cross-sectional online survey on attitudes and practices towards the COVID-19 pandemic [20]. Participation in a follow-up interview was offered to those who had expressed interest and who had reported having a chronic illness. The University of Queensland Human Research Ethics Committee provided approval (#2020000800) and all researchers gained reciprocal approval from their own institutions.

Interviews occurred during the period August to December 2020 and were completed via telephone or video conference and audio recorded. Participants (P) were ascribed a unique identifier indicating their interview number_sex_age_MMM-location where MMM is their Modified Monash Model location, a recognised Australian measure of geographical remoteness and population size (rated 1 through 7 where MMM1 is a major city and MMM7 is very remote) [22].

As per the protocol, interviewers notated a coding table and provided field notes and the audio recording following each interview. The coding table was set out in time intervals and included a range of pre-determined topics or tags likely to come up during the interview. The time intervals allowed the interviewer to note the timepoint in the audio where the topic was discussed. The notes ensured that the interviewer provided reflective discussion points for guidance as per the RITA approach [21]. Audio recordings and notated coding tables were uploaded to a cloud-based shared digital research notebook.

### 2.3. Data Analysis

Analysis using the RITA technique [21] for the broader research project has been previously described [20]. The data set comprised 90 audio recordings and each recording’s corresponding field notes and coding table tagged with topics relevant to the questions being asked.

The analysis was a five-step rapid analysis and coding process undertaken after data collection was complete. The first step was a reflective discussion by the research group which focused on the interviews and identified potential themes for analysis relevant to the research questions.

Steps 2–5 were undertaken by the author team. In Step 2, the author team reviewed the data set to identify interviews and comments related to the research question and commenced creation of a coding system. In Step 3, authors (NC, AT, LT, ST, and LB) reviewed identified interviews and populated a coding form containing participant unique identifier, notes, illustrative quote, emergent theme. The completed coding forms were shared with the author team for review of the themes and agreement on the next steps for coding refinement. In Step 4, two authors (NC, LB) reviewed all completed forms, confirmed the codes and refined the definitions of the themes. Where ambiguity in the applicability of the data to the theme was identified, audio recordings were reviewed to ensure accurate interpretation and to confirm quotes. In Step 5, all authors reviewed the coding definitions and the final coding form containing the assigned codes and relevant quotes. Discrepancies were discussed within the team to confirm meanings and to reach a consensus on interpretations and assignment to themes.

## 3. Results

A total of 90 participants were interviewed comprising 70 women, 20 men: mean age 48 years, (range 20–81 years). Participants were distributed across all states of Australia, and rurality classifications, MMM 1 (*n* = 12), MMM 2 (*n* = 35), MMM 3 (*n* = 7), MMM 4 (*n* = 2), MMM 5 (*n* = 15), MMM 6 (*n* = 10), MMM 7 (*n* = 9). Interviews were approximately 30 min in length. Analysis revealed five broad themes which captured the positive elements respondents described as part of their experience of the pandemic: safety and security; gratitude and appreciation; social cohesion and connections; the opportunity to reset priorities and overall resilience.

### 3.1. Safety and Security

Participants commented on how the travel and border restrictions provided safety and security, particularly in relation to elsewhere around the world, as was evident in the media. They used phrases such as “the borders closed early enough” (P47_F_60_1), “privileged” (P16_F_64_5) and “lucky” (P79_F_43_5) to compare the Australian experience of the pandemic with the situation in other countries.

Additionally, a number of participants noted that Australia’s land mass, urban design and regional centres meant people naturally had space, backyards and distance from each other, “we’ve got more room” (P75_F_69_2), making policy adherence easier.

The messaging around hygiene, the implementation of public hand hygiene stations and physical distancing was felt to have impacted on public perceptions of appropriate hygiene. Participants noted the increased hygiene as reducing colds and flu (“hygiene for all viruses not just COVID” P15_M_67_1) and increasing disease awareness (“not transmitting illness” P27_F_38_2), all contributing to a sense of enhanced community safety and security. As a result, many participants expressed hope that people would continue to take hygiene seriously.

*It’s been an education for us, you know, that we shouldn’t be so complacent with even the flu. People were quite happy to give someone else the flu before…it has taught them that this isn’t the case, because if you do transmit it, it could be fatal for your grandparents, or your parents or someone’s parents or friend of your parents.* (P38_M_64_2)

### 3.2. Gratitude and Appreciation

Participants expressed gratitude and appreciation in several areas. Many talked about how the policy of travel restrictions had opened up telehealth and more accessible health care, providing examples such as convenient access to prescriptions and flexibility for people working full time. People who lived in more remote regions noted that telehealth reduced the tyranny of distance and their need to travel. They described the changes using words such as “impressive” (P70_F_24_2), “important” (P13_F_47_7) and “significant” (P29_F_45_1).

*It’s good to just have that phone consult, fax it off to the nearest pharmacy that we go to, yeah it just makes it so much easier, way easier*. (P1_F_34_5)

Gratitude was expressed for being able to maintain employment by working from home. Participants reported feeling this provided a message to businesses that flexible work environments such as working from home could still be “efficient” (P34_M_47_5) and “productive” (P29_F_45_1) despite previous reluctance to adopt it. Participants in regional and remote areas acknowledged the importance of these work environments both for their communities and for individuals, potential “career opportunities that don’t require re-location” (P10_F_36_7) to larger centres.

*Everyone’s realized not everyone needs to be living in a big city…like I’m hoping that maybe that might allow for more regional jobs where you can access stuff through the internet*. (P50_F_37_3)

### 3.3. Social Cohesion and Connections

Positive impacts on social cohesion and connections emerged as a key theme. There was an increased awareness of the importance of others, technology as a medium to connect was valued, and ironically, the common experience of isolation provided a sense of “coming together” (P82_F_61_6).

The restrictions and lockdown experienced by many participants resulted in an increased consciousness and “valuing” of (P42_F_55_2), and “empathy” for (P24_F_54_2) people around them. Gestures of support from employers were appreciated and confirmed the importance of the participant’s work role. Several participants expressed hope that schoolteachers would be viewed in a positive new light.

*When you have to homeschool youngsters, it’s a very tricky thing to do—so a new respect for teachers has come into play*. (P6_F_68_6)

Existing family and social connections became more valued. At the household level, many remarked that being forced into isolation with family increased their feelings of connectedness. People observed that there seemed to be more time which resulted in feeling reconnected with the family.

*The family was playing card games, interacting a lot better when watching movies together… So I think that is a positive thing from a family perspective…It was kind of forced upon you*. (P15_M_67_1)

The restrictions were reported to increase communication with family and friends, even with people living far away. The slowdown prompted deeper conversations, Zoom parties and video calls. As one participant commented “we spoke more than we normally would” (P43_F_48_5). Many reported checking in on older relatives or known friends who were isolated, alone and/or were thought to “not be doing well” (P58_F_30_4).

Technology was the enabler of connecting outside the household. There were reports of increased text messaging, and video conversations with family and friends, using apps such as Facetime. Live video communication such as Zoom became a common daily occurrence with activities including work and religious ceremonies moving to virtual platforms. For working people, it was felt to be a positive influence on better communication with colleagues without concerns about disturbing others in open plan offices. One participant also remarked that the experience increased connection and engagement with work colleagues because of the personal insights and context gained through “seeing into colleague’s homes” (P54_M_69_2).

Overall, participants experienced a renewed sense of community and togetherness. Examples included interactions such as socially distanced exercise with neighbours, and community teddy bear and rainbow walks for children. People also reported efforts to support local business such as take-away food (P50_F_37_3).

People remarked about having “trust” of the community, feeling “safe” because of the community and feeling “supported”. The shared experience introduced a new “common conversation point” (P57_F_NR_2).

### 3.4. Opportunity to Reset Priorities

Many participants were appreciative that COVID-19 had effectively pushed pause in the demands of their busy life “less pressure, so slowing down has been positive” (P33_F_56_6), “really enjoyed time to take a chainsaw to the garden” (P65_M_65_5). Reassessing their values around what matters in life occurred in part because they had the time to consider this and there was a greater appreciation of others, the fragility of life and the reality of their own mortality (P24_F_54_2; P32_M_69_2).

Words and phrases such as “reset”, “slowed down”, and concepts related to re-evaluating priorities arose in many interviews. Participants reported examples of how this re-evaluation had positively impacted their work-life balance and their commitment to a healthier lifestyle using exercise and cooking from scratch as examples. The re-set was regarded as positive for children because it increased family interactions, and one participant (P50_F_37_3) commented positively that the pandemic had also pressed pause on some children’s extra-curricular schedules.

While people commonly reported that they missed the opportunity to be with family, one participant with cancer (P16_F_64_5) commented that she had appreciated having less pressure to socialise and keep up with family and social commitments, “no need to make excuses”. Not being an extroverted person, she had found that social situations “take a toll”. The respite also presented the opportunity to take up home-based hobbies, enabling her international collaboration on art projects.

Some participants commented positively about the environmental impacts that had derived from less international and domestic travel and the slowdown in some industries. They saw this as beneficial to the planet, an unanticipated sustainability outcome. As stated by one participant “[I’m] grateful that nature got a breathing space. More birds and creatures, things looking less worn out. Quieter due to no planes” (P16_F_64_5). The sentiments were echoed by many others reiterating that this challenging time enabled the rethinking of priorities and a more sustainable approach to life, it enabled steps towards *‘healing the world’* (P33_F_56_6).

### 3.5. Resilience: Finding Strength

Participants who felt they were resilient and had coped well discussed attitudes and behaviours that influenced their healthy functioning during the pandemic. As described in the theme of gratitude, focusing on the positive aspects of the situation was a primary strategy for building strength. However, many participants expanded beyond gratitude to describe a range of attitudes and behaviours towards the pandemic and its management that influenced their mental health. These included acceptance, active resistance of negativity, and intentional adaptability and making good in difficult circumstances.

Acceptance was described using words such as “went with the flow” (P48_F_59_5). Participants recognized that acceptance could help them meet the challenge of uncertainty: “You can’t do anything about that, you just have to accept it…that’s just life”. (P46_F_65_5). Actively resisting negative thinking was described using phrases such as “not allowed fear mongering to get me” (P17_M_61_1) and being “strong enough” (P55_F_59_7). Some also expressed surprise at learning about their mental strength: “I am stronger than I probably thought”. (P86_F_65_2).

Accepting the circumstances, but also being as proactive as possible, was another strategy that participants adopted for coping with the challenges of uncertainty and change: “adapt and keep a positive attitude” (P75_F_69_2). One participant (P26_M_28_7) described a sense of empowerment from their contribution to workplace and community management of the pandemic, while others simply talked about doing “the best they can” (P70_F_24_2).

A range of examples were provided of how people had proactively adapted to ways of exercising, connecting with family and friends, and finding effective work and leisure options. These ranged from starting new hobbies and developing new habits to becoming more personally efficient and adapting to working online. For some, adaptability was a process, not initially evident but took time to develop: “My routine was lost a bit but I’m quite resilient. I worked out a regime at home…that’s worked out quite well” (P62_F_58_3).

Finally, there was a sense of realism that while many individuals could manage their own response and behaviour and experienced that as a silver lining, others had suffered hugely in pandemic (it wasn’t great for some P43_F_48_5) and that the positives might not outweigh the negatives (P29_F_45_1) either for individuals or the community. One participant summed this up as: “I’m lucky in so many respects despite experiencing strong mental anguish” (P60_F_75_3).

## 4. Discussion

This study provides evidential insight for planning public messaging and mental health interventions in future lockdowns and restrictions. While some research has reported negative emotions and risks to mental health [8,9], our findings of silver linings to the early stages of the pandemic aligns with other work demonstrating human tendencies to adapt and notice positives [23,24]. Despite the turmoil resulting from the pandemic’s global spread with its challenging personal impacts across health, social and economic domains, many of the Australians in this study found silver linings in their experiences and hoped that some changes would endure even after COVID-19 was controlled.

The silver linings were articulated despite participants having self-identified as having a chronic illness, which, based on understanding at the time, placed them at greater risk from COVID-19 infection. The five main themes that arose describing silver linings revealed the human capacity to live with uncertainty, loss and unknowns, whilst staying positive and showing overall resilience. Our findings align with finding from a Polish survey study where 65% of participants were able to provide positives during the early stages of the pandemic [25].

Resilience was evident in participants as they spoke about their ability to adapt and be creative in finding new and different ways of coping with life during the pandemic. Importantly, some participants viewed themselves as naturally possessing adequate coping skills, while others found strength and resilience that they had not necessarily expected. Resilience has a fundamental role in sustaining individual healthy functioning, and is implicated in predictions of depression, anxiety and social isolation [26,27]. Challenges can prompt reflection and reassessment of values and priorities, and this was evident in our findings. Public health messaging to promote stories of hope, strength, and showcase ways of coping that sustain individuals through difficulties are needed and can include broad public health approaches such as the importance of exercise as a strategy to build mental health [25].

Our results supported previous findings that the action and connection of individuals with their networks contributed to social and community resilience [28]. Sharing of positive life experiences has been linked to increased positive emotion and to happiness [28]. Efforts to mobilise communities to enable supportive networks during pandemics is likely to be a beneficial public health strategy. As evidenced during the Ebola outbreak, actively engaging with communities in a bottom-up approach gave people a sense of control over their lives despite the uncertainty of the situation [29]. Our participants also voiced a sense of success in knowing that the community could work together and pride that people were advocating or showing concern about others who were vulnerable in the pandemic. Community action plays a vital public health role during a pandemic where hygiene and social distancing are critical, and efforts will need to be sustained for some time.

Community resilience is a dynamic process, with strong social support networks and prior experience with adverse events strengthening the development of collective identity and resilience over time [26,27]. Acts of community service and solidarity among community members are key ingredients in pandemic efforts as they support individual wellbeing as well as contribute to building resilience at the community level. The needs of people living alone or with limited social support networks require consideration in future pandemic preparedness. The implementation of clustering contacts beyond the immediate household, commonly referred to as ‘social bubbles’, is an effective strategy to increase social contact while limiting associated epidemic risk [30]. Community programs that focus on supporting isolated individuals to maintain connected during pandemic lockdowns are also important.

The global geographical isolation of Australia, as well as the remoteness of cities and communities within the country have sometimes been seen as a disadvantage for Australians with an interest in travel or commerce. However, participants recognised Australia was fortunate for its geographical isolation and knew that comparatively they were living in privileged circumstances. They acknowledged that Australian border controls, infrastructure, geographic isolation and government policy had all created a bubble of safety that was largely containing entry of the COVID-19 virus and minimising outbreaks in Australia. In addition and aligned with commentary by Manzanedo and Manning [31], participant observation of the unexpected positives for global and local sustainability hints at public appetite for approaches for future policy addressing climate change.

Appreciation of the public health measures which were protecting Australians from the devastating impacts of the disease observed overseas was evident [4,32]. However, our findings suggest that public messaging could draw more upon community voices to emphasise the silver linings of Australia’s public health response, including the benefits of connectedness despite physical separation and opportunities for a more environmentally sustainable future.

The challenges arising from this global pandemic have placed a great demand on the mental wellbeing of Australians in multiple ways. In the last half of 2021, many Australians found themselves facing a new wave of lockdowns, restrictions, and uncertainties with clear evidence of heightened population concern and psychological distress. Responses received in our study reflect a point in time and not the impact of ongoing uncertainty that the pandemic has created. However, awareness of perceived silver linings, particularly those related to social cohesion, safety and security and others influencing perceived agency are important to incorporate and prioritise into public health strategies as the country responds to the evolving pandemic. People with chronic disease are more vulnerable to mental health issues and there is a growing body of literature on mental illness in people with chronic disease during the pandemic [33,34].

It is acknowledged that many Australians have suffered deeply due to the impact of the pandemic. No information on the mental health of the individuals interviewed was specifically sought however it should be noted that out of 90 interviews, only six definitively stated that they could find no positives through the experience. This large proportion of respondents who noted positives suggests the tenacity of the human spirit in facing adversity and “making the best”. It is important however that policy and support services consider the needs of people for whom the mental health stresses far outweigh the ability to focus on positives.

### Limitations

The use of rapid identification of themes from audio recordings (RITA) technique enabled the research team to work with a large number of interviews without line-by-line coding and analysis. The rigour of the process as described in the methods section enabled efficiency and cross-checking to obtain consensus on the coding. The trade-off in using the RITA technique was that the efficiency gains from reliance on the field notes and time-marked coding tables (see Section 2.3) may have resulted in less nuance of individual participant’s experience while affording a comprehensive picture across participants.

The cross-sectional study does not capture changes over time, the evolving and changing nature of COVID-19 experiences, which undoubtedly would impact perceptions of positive aspects. However, the snapshot of this period can help our understanding of the reaction and thoughts of people living through a global pandemic.

## 5. Conclusions

By examining the silver linings expressed by Australians during a period of significant public health restrictions and considerable uncertainty from COVID-19, this study has demonstrated the remarkable ability of people to express positivity and overall resilience in the face of adversity. The study confirms that, at least for some, the response to the COVID-19 pandemic provided an opportunity to reflect on and reassess their values and priorities—what is important for them, their families, and their community. These findings provide a unique perspective when considering the priorities of Australians and the public health implications for a post-pandemic society.

## Data Availability

The data is stored in a secure digital repository through The University of Queensland. Approval to access to the repository can be sought through Geoff Argus on g.argus@uq.edu.au.
